# Supplementary material for: Bone Marrow-Derived Mesenchymal Stem Cell Implants for the Treatment of Focal Chondral Defects of the Knee in Animal Models: A Systematic Review and Meta-Analysis
Source: Int J Mol Sci. 2023 Feb 6;24(4):3227. doi: 10.3390/ijms24043227 (PMC9958893; doi:10.3390/ijms24043227)
Supplement: Supplementary file 1 [file ijms-24-03227-s001.zip › Supplementary Figure S1.pdf]

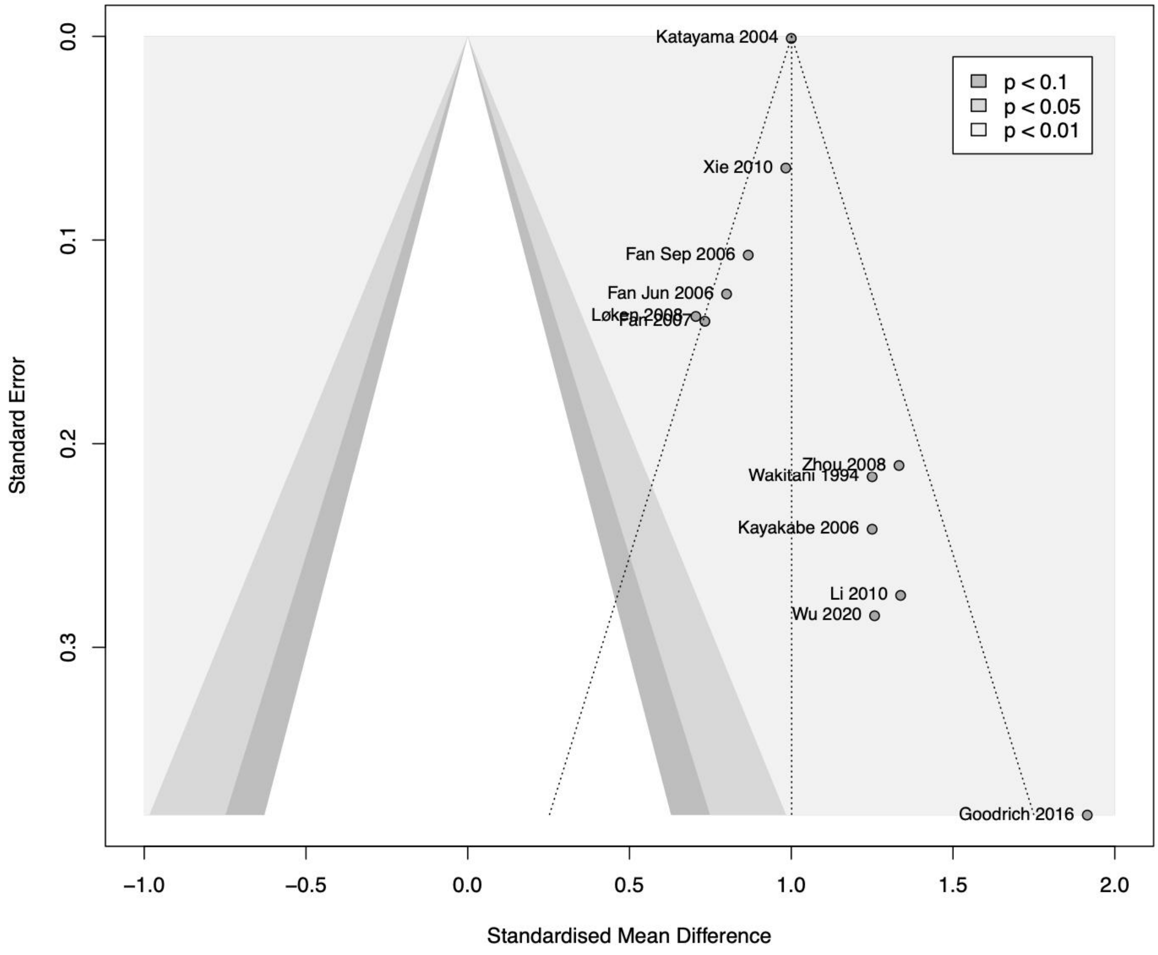

Egger's test of the intercept for publication bias: $p = 0.82$.
Egger's test does not indicate the presence of funnel plot asymmetry.

Supplementary Figure S1. Contour-enhanced funnel plot for publication bias.
