# Supplementary material for: Bone Marrow-Derived Mesenchymal Stem Cell Implants for the Treatment of Focal Chondral Defects of the Knee in Animal Models: A Systematic Review and Meta-Analysis
Source: Int J Mol Sci. 2023 Feb 6;24(4):3227. doi: 10.3390/ijms24043227 (PMC9958893; doi:10.3390/ijms24043227)
Supplement: Supplementary file 1 [file ijms-24-03227-s001.zip › Supplementary Table S1.pdf]

Supplementary Table S1. Detailed search strategy.

Knee	Cartilage variations	Cartilage to implant variations	Defect variations	MSC variations	Implant variations	Investigation variations	
1) Knee	2) Chondral<br>3) Cartilage<br>4) Articular<br>5) 2 OR 3 OR 4	6) Cartilage-cartilage<br>7) Cartilage to cartilage<br>8) Cartilage-implant<br>9) Cartilage to implant<br>10) 6 OR 7 OR 8 OR 9	11) Disease<br>12) Defect<br>13) Damage<br>14) Lesion<br>15) 11 OR 12 OR 13 OR 14	16) Mesenchymal stem cell<br>17) MSC<br>18) (BM or bone-marrow or bone marrow) derived<br>19) Adipose-derived or adipose derived or ASC<br>20) (Blood or peripheral blood or peripheral-blood or PB) derived<br>21) 16 OR 17 OR 18 OR 19 OR 20	22) Implant<br>23) Scaffold<br>24) Graft<br>25) Inject<br>26) 22 OR 23 OR 24 OR 25	27) Interface<br>28) Integration<br>29) Histology<br>30) Arthroscopy<br>31) MRI or magnetic resonance imaging<br>32) 27 OR 28 OR 29 OR 30 OR 31	33) 1 AND 5 AND 10 AND 15 AND 21 AND 26 AND 32
